# Programmable LED Array for Evaluating Artificial Light Sources to Improve Insect Trapping

**DOI:** 10.3390/insects16020170

**Published:** 2025-02-06

**Authors:** Mohsen Paryavi, Keith Weiser, Michael Melzer, Damon Crook, Chandrika Ramadugu, Daniel M. Jenkins

**Affiliations:** 1 Department of Electrical & Computer Engineering, University of Hawaii, Holmes Hall 483, Honolulu, HI 96822, USA; mparyavi@hawaii.edu; 2 Department of Plant and Environmental Protection Sciences, University of Hawai’i at Manoa, 3190 Maile Way Room 305, Honolulu, HI 96822, USA; crbops01@hawaii.edu (K.W.); melzer@hawaii.edu (M.M.); 3 United States Department of Agriculture, Animal and Plant Health Inspection Service, Plant Protection and Quarantine, 1398 West Truck Road, Buzzards Bay, MA 02542, USA; damon.j.crook@usda.gov; 4 Department of Botany and Plant Sciences, University of California Riverside, Riverside, CA 92521, USA; chandrika.ramadugu@ucr.edu

**Keywords:** coconut rhinoceros beetle, Asian citrus psyllid, light preference, pest delineation, integrated pest management

## Abstract

We developed a programmable LED array to evaluate different wavelength illumination (UV, blue, green, yellow, amber, and red) and modulation schemes to improve catch rates in insect traps. The device can communicate through Bluetooth^®^ with a simple Android app to update the operational settings to facilitate field experiments, including which LEDs to operate, when to operate (always, night only, or predefined intervals after sunset and/or before sunrise), and to change the LED intensities/modulation during operation. We used the devices to evaluate different wavelengths to improve catches in traps for coconut rhinoceros beetle (CRB; *Oryctes rhinoceros* Linnaeus) in the field, as well as to evaluate lighting preferences of Asian citrus psyllid (ACP; *Diaphorina citri* Kuwayama). In both cases, insects were most strongly attracted to constant UV illumination. However, CRB avoided traps with any “visible” wavelength LEDs placed in panels of traps, while ACP was moderately attracted to blue, yellow, and amber. For CRB, UV illumination of cups at the bottom of panel traps reduced catch rates compared to UV illumination higher in the panels of traps, consistent with observations of dorsal orientation towards light observed by other researchers in nocturnal beetles and moths. Finally, we provide some hardware design recommendations to improve the energy efficiency of similar devices for more widespread deployment in insect traps and for controlling the LEDs to evaluate the effects of intensity and modulation with minimal pulsing, which our observations suggest may result in insects avoiding traps.

## Simple Summary

Insect monitoring is critical for implementing effective pest control that minimizes harm to people and the environment. Artificial lighting is often used to improve insect trapping, especially for nocturnal insects. Most insects have a trichromatic color vision system, with UV, blue, and green photoreceptors, though many also have sensitivity well into the red portion of the spectrum. We developed a programmable LED array with wavelengths spanning those perceived by insects to facilitate field experiments to identify specific lighting conditions to improve insect trapping. The LED array is programmable through a graphical user interface in an Android app using wireless communication. Researchers can swiftly program the operating parameters including wavelength selections with intensity and modulation, and timing of operation throughout the day. We deployed the devices in traps for two important insect pests, coconut rhinoceros beetle (CRB) and Asian citrus psyllid (ACP). Our results indicate that both species were significantly more likely to be caught in traps illuminated with unmodulated UV compared to other wavelengths or unlit traps. In contrast, catches in CRB traps illuminated with any visible (non-UV) color LEDs were significantly diminished compared to traps without illumination.

## 1. Introduction

Identification, delineation, and enumeration of insect populations is a pillar of integrated pest management [1,2,3]. Currently, there are about 1800 pheromone-baited panel traps for coconut rhinoceros beetle (CRB; *Oryctes rhinoceros*) distributed throughout the island of Oahu to delineate progress of an invasion first reported in December 2013 [4], and additional traps are being deployed to the islands of Maui, Hawaii, and Kauai where invasions of the beetle were discovered in 2023. Instrumentation of these traps is especially critical for early detection and control operations on the latter islands, with the first identification of an adult CRB on the island of Hawaii occurring in a trap with a remote surveillance system [5]. Control of CRB is a high priority in the state of Hawaii and other Pacific Islands due to the threat they pose to both cultivated and indigenous palm trees in natural areas and various food crops. Control is essential near ports to prevent the spread to other islands or commercial palm operations in other areas of the United States such as southern California. Trapping is also a critical component of IPM for the Asian citrus psyllid (ACP, *Diaphorina citri* Kuwayama), the insect vector of the bacterial disease huanglongbing (HLB) in citrus. In the United States, the pathogen has devastated the citrus industry in Florida, has spread to every citrus-producing state except Arizona and Hawaii, and poses a significant threat to the citrus industry in California [6]. Keremane et al. [7] demonstrated that molecular detection of the HLB pathogen in ACP could be used for successfully identifying infected trees years before the emergence of symptoms or the ability to detect the fastidious pathogen in the plant material directly. Therefore, extensive research has been dedicated to developing effective ACP traps from which genetic material can easily be recovered [8,9] and rapid diagnostic approaches for the HLB-associated bacteria [6,10,11] to help mitigate the disease in California. Area-wide management of ACP is considered essential to control the huanglongbing disease of citrus in California; significant resources are allocated for psyllid trapping and control to minimize disease spread and protect the USD 3.63 billion citrus industry in California [12].

Phylogenetic and molecular analyses suggest that most insects have evolved a trichromic color vision system with UV, blue, and green receptors and that a few minor variations are primarily due to adaptations to visual ecology [13,14,15,16,17]. Virtually all insects characterized have peak spectral sensitivity of their first photoreceptor in the UV range between 350 and 400 nm, and numerous species have been described, with up to six or more photoreceptors sensitive well into the red portion of the spectrum [18]. In contrast with most vertebrates, several nocturnal insects have been shown to have photoreceptors sensitive enough to discriminate color in dim nighttime conditions to differentiate flowers [17] or fruit [19]. Dacke et al. [20] showed that some crepuscular dung beetles have receptors adapted to the detection of dim polarized light, with field research demonstrating that these beetles use the polarization of moonlight through the atmosphere for orientation and navigation [21]. Yilmaz et al. [22] demonstrated that the perception of polarized moonlight is most acute in the green photoreceptors of these beetles. Sensitivity to polarized light has also been described in several aquatic insect species [23], and contamination of the environment with artificial light or surfaces that enhance light polarization can disrupt appropriate habitat selection [24,25,26]. Navigation and orientation through cues from celestial light are almost universal in insects [23,27,28,29,30]. Fabian et al. [31] used high-speed cameras to record flight trajectories and orientations of a variety of nocturnal insects flying through artificial light, demonstrating that insects exhibited a “dorsal tilting” towards the light source, supporting the hypothesis that insects use celestial light sources to maintain stable flight attitude in nature.

A significant amount of effort has been made to use color and lighting to improve insect trap performance, including through direct evaluation of different color materials, lights, and unique illumination patterns of traps in field trials [8,9,32,33], or through electrophysiological evaluation of insect vision [34,35,36] to craft palettes with spectral reflection or lighting more closely tuned to insect visual experience [14,16,35]. Artificial lighting is prevalently deployed to enhance the capture of insects, especially those active nocturnally, both for general ecological censuses and for IPM. In these scenarios, lighting with a significant UV component generally performs best (i.e., [32,33]). Robertson and Horvath [26] demonstrated that nocturnal aquatic insects are not preferentially attracted to horizontally polarized lamps but generally avoid red and blue light. Kamei et al. [37] observed that white LEDs (with emission bands primarily in the visible spectrum) were less effective for attracting insects than Mercury Vapor fluorescent lighting with strong spectral lines in UV and purple, though for some orders of insects, this difference was mitigated by using white LEDs with a more “cool” (i.e., blue) characteristic. Komatsu et al. [38] deployed lighted insect traps during summers in Northern Japan to determine that some aquatic insects and winged ants were attracted to a broad spectrum (UV-A to green), whereas moths and beetles were more attracted to a narrower spectrum of wavelengths (UV-A to blue). Similarly, Al-Deeb [39] showed that palm-infesting beetles similar to CRB were more attracted to mercury vapor lamps with significant short wavelength radiation than they were to lights with radiation in the longer wavelength visible parts of the spectrum.

To evaluate different color LEDs for improving the catch rates in traps for CRB and ACP, we developed a custom programmable LED array (with center wavelengths of 365 nm, 385 nm, 460 nm, 530 nm, 585 nm, 610 nm, and 620 nm), where each LED could be illuminated independently with a variety of modulation schemes and at different times of day relative to perceived sunset and sunrise times. In this manuscript, we provide a cursory technical description of the lights with more detailed fabrication files, a bill of materials, the source code, and the device manual, which are included as Supplementary Materials. We also share the outcomes of various experimental trials with the lights to evaluate the effects of artificial lighting in traps for CRB and ACP.

## 2. Materials and Methods

### 2.1. Hardware Design

**Figure 1:**
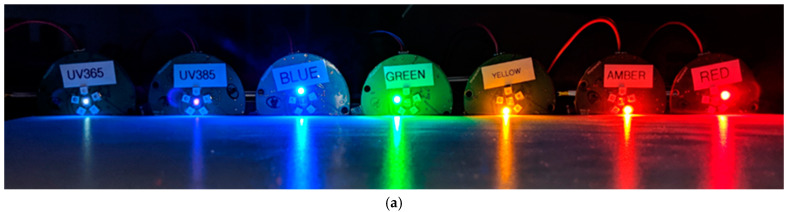

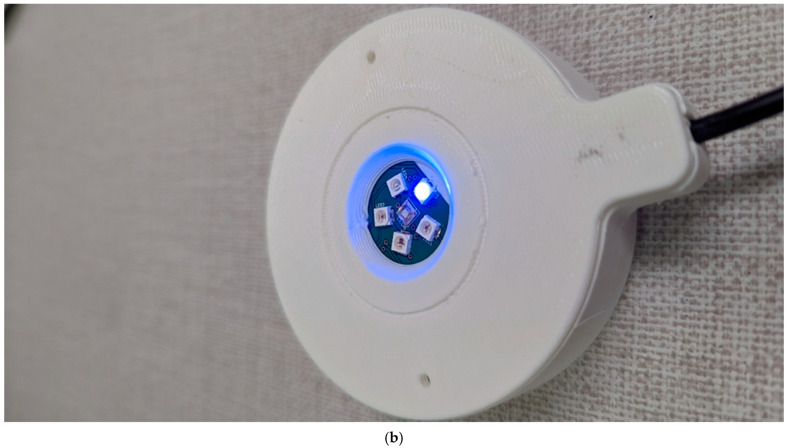
(**a**) Programmable LED arrays with different colors illuminated; (**b**) a custom enclosure for field trials in panel traps for CRB, without the attached diffuser (shown with a blue LED illuminated).

Programmable LED arrays (Figure 1a; fabrication files and other details shared through the Supplementary Materials) were built around the ATMEGA328P microcontroller (Microchip Technology, Chandler, AZ, USA) clocked with the internal 8 MHz oscillator and powered by a 3.3 V switching regulator (TPS63031, Texas Instruments, Dallas, TX, USA) from a single-cell LiPO battery charged through a linear lithium battery charging chip (MCP73831, Microchip Technology). An array of 6 LEDs (UV, blue, green, yellow, amber, and red) were each independently controllable through separate PWM outputs gating individual linear LED drivers (AS1102-T, ams-OSRAM USA, Wilmington, MA, USA), nominally set to output 84 mA when on. LED currents were sourced from switching regulators, one nominally outputting 5.5 V (TPS63060, Texas Instruments) to overcome the higher forward voltages for the UV, blue, and green LEDs, with the remaining LEDs powered from the 3.3 V used for the microcontroller. Two variants of the device were made with slightly different UV LEDs (365 nm or 385 nm; LTPL-C034UVH series, Lite-On Inc., Milpitas, CA, USA), while the same series of “visible” color LEDs were used for blue, yellow, amber, and red illumination in each device (OVS5M Series, “blue”/460 nm, “yellow”/585 nm, “amber”/610 nm, and “red”/620 nm, TT Electronics/Optek Technology, Woking, UK). Green LEDs were sourced from a different manufacturer (L135-G525003500000/530 nm, Lumileds, San Jose, CA, USA) due to non-availability of the green variant of the Optek LED when the devices were manufactured. To facilitate wireless field programming to set parameters such as selecting which LED(s) to operate under which modulation scheme and durations of operation, a serial Bluetooth module (RN42, Microchip Technologies Inc.) connected to the microcontroller can physically be powered through a jumper from the 3.3 V regulator. A spring-loaded terminal was included to plug in a 33 kΩ nominal photoconductive cell (PDV-P8103, Advanced Photonix, Inc, Ann Arbor, MI, USA) to roughly distinguish day from night by measuring the 3.3 V from a digital output divided between the photocell and a 27 kΩ resistor. For the field trials described here for CRB, we 3D-printed custom enclosures for the lighting devices, using thin-walled “glow in the dark” ping pong balls (Kolorae Brand, Blueoco, Grandville, MI, USA) cut into hemispheres glued around large orifices below the LEDs to act as an omnidirectional light diffuser (Figure 1b). Recorded spectra for each LED (test setup shown in Supplementary Materials Figure S1) with and without the diffuser is shared in the Supplementary Materials (Figure S2; Table S1), with results conforming to the well-known “green gap” where LEDs in the mid-visible wavelengths are prone to lower quantum efficiencies than blue and red LEDs [40,41]. The data also illustrate that the diffuser did not appreciably alter the spectra of light from any LED and that it was effective at scattering light so that the device could be seen from any side (e.g., Supplementary Materials, Figure S1). Even so, the shorter wavelength LEDs were subject to higher transmission losses through the diffuser compared to the green, yellow, amber, and red LEDs.

### 2.2. Software Design

The firmware on the LED array was developed using the Arduino IDE (Arduino, Somerville, MA, USA), with a custom companion app for field programming developed in Android (Google, Mountain View, CA, USA). To save energy when the LEDs are not in use, the microcontroller stays in a low-power idle mode for increments of one minute and, when awaking from the idle mode, determines whether the lights should be operated based on the desired time-of-day settings (always on, night only, or defined intervals after sunset and/or before sunrise), activating the photosensor circuit to measure ambient light to predict whether it is day or night. Consecutively observed night minutes are counted and recorded by the microcontroller to predict the time difference between sunset and sunrise for the subsequent day and correctly time desired lighting with respect to sunrise. Several minutes are required with consistent perceived lighting conditions to count a change to or from night to prevent disruption of timing by spurious/transient lighting events, such as during servicing of the lights. The device firmware and Android installation file for the app, along with a manual for operating the system, is shared in the Supplementary Materials.

### 2.3. Artificial Lighting Effects in ACP Traps

**Figure 2:**
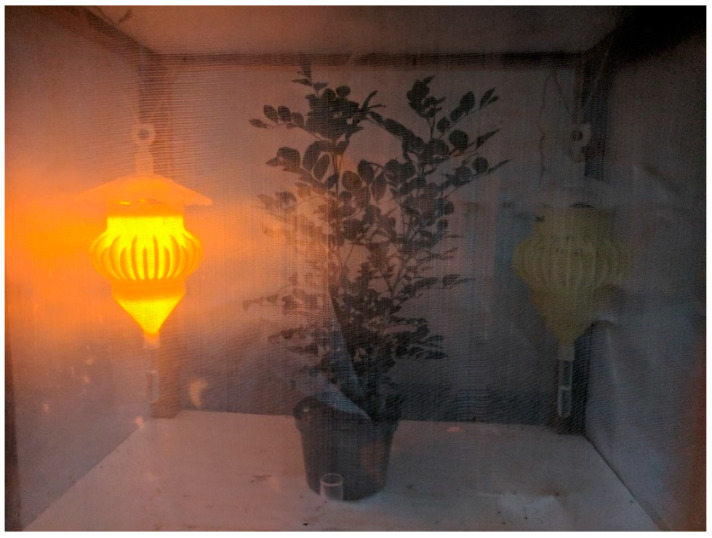
Setup for forced-choice experiments to evaluate lighting preference of Asian citrus psyllid (*Diaphorina citri*).

To evaluate artificial lighting for attracting ACP, we placed LED arrays in the “tripod structure” in the top of custom-designed “stem traps” for ACP [8]. The main 3D-printed body of these traps includes an array of 16 curved “stems” that pass through holes into the inside of the trap body, inspired by observations that ACP walk along stems of host plants and hypothetical phototaxis towards the holes in the body due to ambient illumination through the transparent rain skirt at the top of the trap. ACP passing into the inside of the trap eventually falls into a tube of preservative fluid (ethanol in the case of these relatively brief experiments). To evaluate attraction to individual wavelengths, we first conducted forced choice experiments with two identical traps and lighting systems, with the selected LED fully illuminated in one trap and the other trap unlit. In these experiments, 20 freshly caught ACP from a heavily infested mock orange (*Murraya paniculata*) hedge were released into the middle of a screened cage (approximating a cube with 1 m sides), with traps suspended on either side of a small potted mock orange plant (Figure 2), repeating each forced choice experiment at least three times. Catches in each trap were recorded after two hours, and “attraction” to the given LED was inferred based on binomial probabilities of the observed results, assuming a hypothesis that both traps were equally likely to attract ACP. To confirm the most effective wavelength for enhancing trap catch, we conducted similar binary forced-choice experiments among the three most “attractive” colors identified in preliminary experiments (i.e., forced-choice experiments with two traps illuminated with different color LEDs), releasing 25 freshly caught ACPs in each trial. To evaluate the effect of LED modulation, we conducted similar experiments (again releasing 25 freshly caught ACPs for each trial) with one trap illuminated fully with UV (385 nm version) and a second trap using modulated UV light, either pulsed fully on and off at a given frequency and 50% duty cycle, or with sinusoidal half waves at the same frequency. The modulation frequency (1.4 Hz) was selected corresponding to a characteristic strong peak in the Fast Fourier Transform (FFT) of light measured with a downward-facing photosensor held in a citrus canopy in bright sunlight (Supplementary Materials Figure S3), hypothesizing that these light conditions would be consistent with those that ACPs encounter in their natural habitat.

A small field trial was also conducted with LED arrays mounted into six separate stem traps hung in a heavily ACP-infested 10 m long section of mock orange hedge on the University of Hawaii at Manoa campus. The lights in five of the traps were programmed to illuminate to approximately 64% intensity with different color LEDs (UV/385 nm, blue, green, yellow, and amber) for 2 h each day, ending near sunrise, and the light in the sixth trap was programmed not to turn on. Traps were rotated through the different stations around the hedge each day after recording the corresponding ACP catch and swapping out the batteries with fully charged 2000 mA-hr LiPO batteries for a total of 6 days (1 day for each trap at each station). Attraction to a specific light treatment (or no light) was inferred from the binomial probability of catching at least as many ACPs as observed for the treatment, assuming all treatments are equally likely to result in ACP capture.

### 2.4. Artificial Lighting Effects in CRB Traps

**Figure 3:**
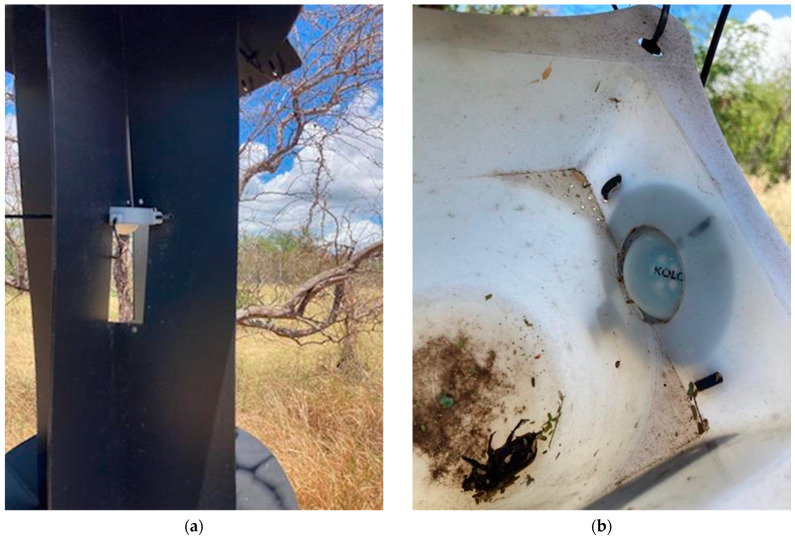
Programmable LED arrays in traps for coconut rhinoceros beetle (*Oryctes rhinoceros*): (**a**) deployed in the panel of CRB panel traps; (**b**) deployed in a cup of CRB panel traps (**b**).

**Figure 4:**
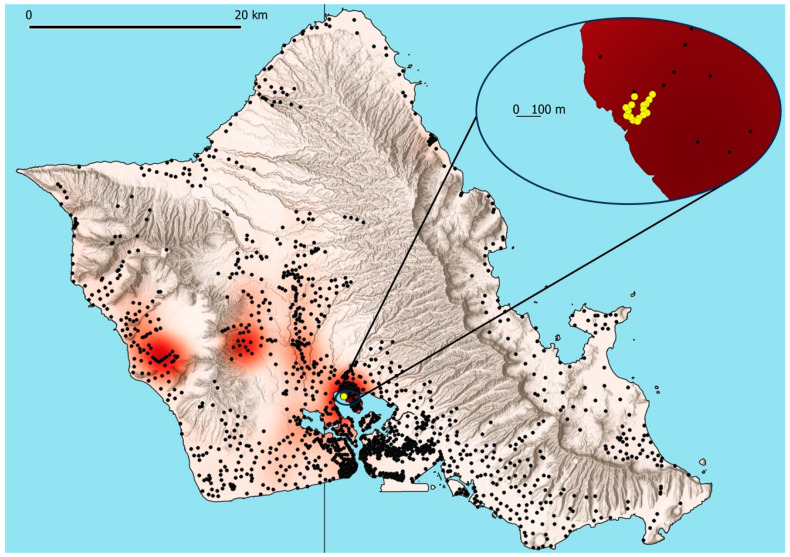
Lighting preference experimental trap locations for coconut rhinoceros beetles (*Oryctes rhinoceros*) and catch heat map (September–December 2023); the heatmap uses default “red” color ramps with 2.5 km radii on QGIS 3.34 for all geolocated panel trap catches on Oahu in September–December (n = 11,716).

To evaluate artificial lighting in traps for CRB, we tested 16 different treatments with the LED arrays in individual panel traps (Panel Trap Black, Alpha Scents, Inc., Canby, OR, USA), each with a pheromone lure (P-O46 Oryctalure, ChemTica SA, Heredia, Costa Rica) changed monthly. For each color (UV 365 nm, UV 385 nm, blue, green, yellow, amber, and red), we programmed two arrays to illuminate that color LED fully (100% duty cycle) for 6 h after sunset and 3 h before sunrise based on the observation that CRB is crepuscular, with more than 2/3 of catches in unlit traps occurring within 3 h of twilight after sunset, and almost all other catches occurring throughout the rest of the night and during twilight [42]. We then placed one array of each programmed color into the panels of individual traps near the pheromone lure with the light oriented downwards (Figure 3a) and the second array of each programmed color on the side of the trap cup of separate traps (Figure 3b). For the control, we used two unlit panel traps. Each trap was also fitted with a remote surveillance system [42] programmed to upload images of the cup contents hourly to verify whether catches occurred while the lights were in operation using the approach described by Paryavi et al. [42] to estimate time of catch relative to sunrise and sunset. Each of the panel traps was rotated on a weekly basis through 16 different trap locations distributed around a roughly 100 m diameter circle near the West side of the Pearl City Peninsula (Figure 4), selected as a relatively undeveloped location with abundant CRB populations, no ambient street lighting, and little human traffic with access controlled by Joint Base Pearl Harbor Hickam. To ensure stable operation of lighting treatments throughout the 16 weeks of the field trial, the lights were deployed with 2000 mA-hr LiPO batteries and USB output photovoltaic panels with 6 W nominal power output (Soshine, Shenzhen, China).

Weekly catch data for each treatment in the CRB field experiments, excluding catches that definitively occurred outside of times that the lighted traps were illuminated based on the time of catch estimated from the remote surveillance system, were evaluated to determine the effects of different LEDs, trap positions, and weeks. As the data did not have the depth to conduct multiway ANOVA for all factors (i.e., multiple replicates of each lighting placement/wavelength treatment for each trap position and weekly interval), the statistical significance of light condition, trap position, and week were evaluated separately using one-way ANOVA (MATLAB 2020B, MathWorks, Natick, MA, USA). To compare individual lighting treatments (7 different colors deployed either in the trap panel or cup, or unlit traps), we evaluated the binomial probabilities of catching at least as many CRBs for the treatment as was observed, assuming CRBs are equally likely to be captured in any treatment, with lower probabilities supporting more effective CRB capture with the treatment. To evaluate treatments associated with depressed catch, we similarly assessed binomial probabilities of catching the observed number or fewer CRBs for a given treatment, assuming that all treatments are equally likely to result in CRB capture.

## 3. Results

### 3.1. Artificial Lighting Effects in ACP Traps

**Figure 5:**
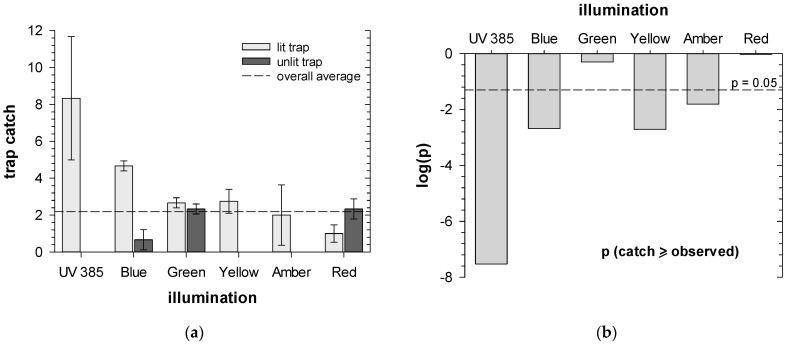
Trap catches in forced-choice experiments in stem traps for Asian citrus psyllid (*Diaphorina citri*): (**a**) showing results of traps lit with different LED wavelengths against unlit traps; (**b**) binomial probabilities of observed treatment catch if lighting has no effect.

Our data show that in forced-choice experiments with fully illuminated LEDs, UV lighting (385 nm) consistently resulted in the highest capture of ACP in stem traps, whether compared to unlit traps (Figure 5; Supplementary Materials Table S2) or in head-to-head experiments with the next best-performing LEDs (blue and yellow; Supplementary Materials, Table S3/Figure S4). ACP capture in traps illuminated with blue, yellow, or amber LEDs was less than with UV but still statistically significant compared to unlit traps (Figure 5). Interestingly, there was no statistical difference observed in forced-choice experiments with traps illuminated fully with UV and traps with pulsed UV at 1.4 Hz with 50% duty cycle. However, there was a strong preference for full UV illumination when compared to 1.4 Hz sinusoidal half wave modulation (Supplementary Materials Table S4, Figure S5).

While higher catch numbers were consistently observed in forced-choice experiments with UV-illuminated stem traps, in field trials comparing daily ACP captures in stem traps illuminated with different LEDs for two hours prior to sunrise, no significant differences were observed for any treatment. In fact, in these field experiments, no lighted treatment resulted in as many ACP captures as was observed in the unlit stem trap (Supplementary Materials, Table S5, Figure S6).

### 3.2. Significance of Lighting, Trap Position, and Weekly Interval for CRB Capture

One-way ANOVA of weekly catches conducted separately for each factor (light treatment, trap position, and week of experiment) indicated that lighting had a statistically significant effect on trap catch in CRB panel traps (*p* = 1.08 × 10^−5^; Table 1) and that there was a marginally significant variation in total trap catches each week (*p* = 0.0445; Table 2). However, the trap locations through which traps were rotated did not exhibit a significant effect on trap catch (*p* = 0.551; Table 3), suggesting that the observed significance of the lighting treatments is unlikely to be merely an artifact of interactions of temporal and spatial variations of CRB catch in the field site used for this study.

### 3.3. Evaluation of Lighting Effects for CRB Capture

**Figure 6:**
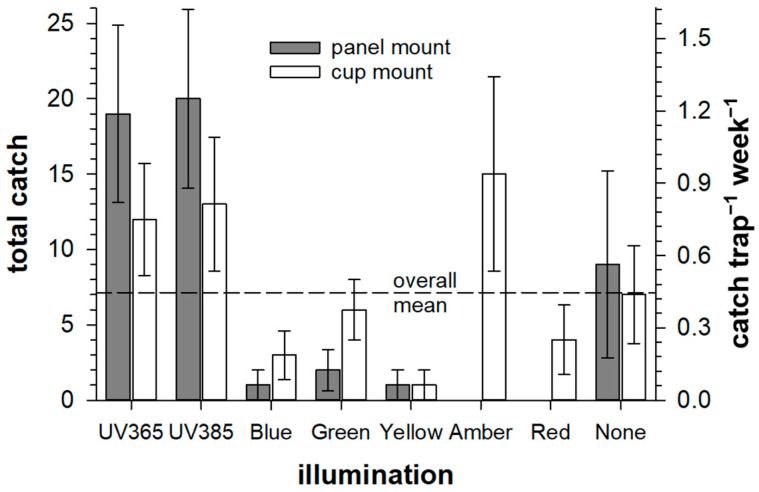
Total trap catches of coconut rhinoceros beetle (*Oryctes rhinoceros*) for each light treatment (first vertical axis), also expressed as a catch rate per trap per week, with error bars equivalent to the standard deviation of the mean weekly catch (second vertical axis).

**Figure 7:**
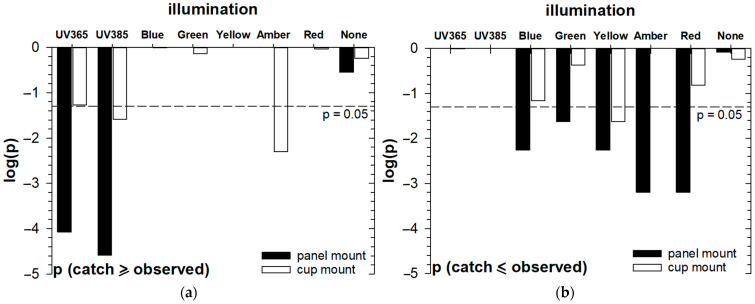
Tests for coconut rhinoceros beetle (*Oryctes rhinoceros*): (**a**) “attraction” and (**b**) “aversion” to different light treatments based on binomial probabilities of total observed catches for each treatment. The results suggest that UV improves trap catch performance for CRB, especially if placed in the panel of traps, and other color LEDs suppress CRB catch if placed in the panel of traps. The observed effects are generally moderated if LEDs are used to illuminate the trap cups.

A comparison of CRB catches for each light treatment (Figure 6) illustrates that more CRBs were caught in traps illuminated with UV LEDs than other treatments and that the unlit “control” treatments had catch rates near the overall mean for all treatments and were, in effect, an appropriate benchmark for evaluating performance with different lights. Using UV illumination at either wavelength (365 nm or 385 nm) in the panel of traps resulted in more than twice the catch rate observed in unlit traps. However, the placement of UV lights in the cup of traps was somewhat less effective for improving catch. The binomial probabilities evaluated for total catches in each light treatment (assuming equal probabilities of catching in each trap) supported a strong preference of CRB for UV illumination in trap panels and a more marginal preference for UV illumination of trap cups. However, there was a significantly increased catch if the cup was illuminated with amber LED (Figure 7a). The last result is especially remarkable, as illumination of the panel of traps with amber did not result in any CRB catches (Figure 6). Tests for aversion (Figure 7b) indicated significant suppression of CRB catches when the trap panels were illuminated with any color other than UV; in fact, no CRBs were caught at all if the panels were illuminated with amber or red.

## 4. Discussion

The results of our experiments were generally consistent with previous work [32,33,37,38,39] suggesting that insects tend to be more attracted to shorter wavelength/UV radiation. Sétamou et al. [43] demonstrated that ACPs are most active in the early afternoon, though the capture of ACP in the field on yellow sticky cards could be improved by illuminating citrus canopies with fluorescent lighting or white LEDs at night. Similar reports indicate that ACP capture is enhanced on yellow sticky cards in shipped fruit when illuminated with white LEDs [44]. In our preliminary forced-choice experiments in dark cages, ACP collected during the daytime exhibited a strong preference for custom stem traps illuminated with UV over unlit traps, with weaker but significant preferences for traps illuminated with blue, yellow, or amber LEDs compared to unlit traps. These results are consistent with the phototactic experiments by Kalile et al. [45], in which color LEDs in the visible spectrum (green, light blue, and red) are equally attractive to ACP, but the strongest attraction was observed for UV light alone or in combination with other wavelengths. Similarly, Paris et al. [46] tested the attraction of ACP to a sticky surface in a small enclosure and observed that illumination through yellow or green filters improved attractiveness, especially if partially illuminated with UV.

While ACP capture was significantly enhanced in UV-illuminated traps in forced-choice experiments, illumination of traps before dawn in field experiments failed to improve the catch rates of ACP. This was potentially due to interferences from strong street lighting over the entire test area, consistent with observations that light-mediated insect trapping has gradually become less effective with urbanization and the proliferation of ambient light pollution [47] and that even fuller lunar phases are associated with suppression of insect catches in illuminated traps [48]. However, the lack of effect for ACP trap lighting observed in field trials may also be consistent with the general inactivity of the diurnal insect [43] when traps were illuminated. The lack of significance of ACP capture in lighted traps in field trials is also consistent with the observed avoidance of ACP of 1.4 Hz “half-wave” modulated UV light, as the modulation was achieved by adjusting the duty cycle of 500 Hz default PWM frequency. A similar strobing would have occurred in the field experiments with ACP, where lights were programmed to only illuminate at 64% intensity. This hypothetical avoidance of insects to the strobing PWM frequency guided the experimental design for CRB field experiments, where illuminated LEDs were programmed to fully turn on with no pulsing.

Our observed results with UV illumination in CRB traps were consistent with those of Siderhurst et al. [33], with UV illumination of vanes/panels of CRB traps resulting in about double the catch rate of unlit traps, though the overall catch rates in our experiments were about twice as high as observed by Siderhurst et al. [33], suggesting a locally higher population density of CRB in our field site. Still, these differences may also be due to differences in the trap design or the greater ability of CRB to escape from the larger buckets used in the earlier study.

Interestingly, CRB catch was most enhanced when UV illumination was used in the upper panel of traps, consistent with the observations by Fabian et al. [31] of the dorsal orientation of nocturnal insects to light sources, which would increase the likelihood of collision with trap panels when lighting is placed higher (i.e., nocturnal insects will fly oriented with lights above their back, such that in ordinary flight, they will be beneath the light source). For UV lighting, the lower effectiveness of lighting in the cup may also have resulted from changes in the UV light spectrum due to diffusion through both the diffuser on the lighting enclosure and diffusion through the trap cup. While longer wavelength LEDs placed on the trap panels resulted in a significant reduction in CRB catch (and no beetles caught with yellow, amber, or red LEDs), the illumination of trap cups with amber significantly increased CRB catch. It is not clear if this occurred due to random chance or if amber caused the CRBs to divert their flight paths upwards away from the light, making it more likely to collide with the trap panel.

While our programmable lighting array facilitated the execution of field trials to quickly evaluate different-wavelength LEDs for attracting insects, specifically CRB and ACP, several hardware limitations precluded deeper investigations into lighting intensity and modulation for improving trap performance or rapid translation to an energy-efficient and affordable lighting module for widespread deployment in traps. For energy efficiency, most off-the-shelf solar LED lighting is based on switched-mode charging of affordable AA Nickel Cadmium batteries and switched-mode current regulation for illuminating the LED from the battery. It is unclear whether our preliminary results showing depression in ACP catch in traps with modulated UV light indicate aversion to slowly varying (1.4 Hz) semi-sinusoidal illumination or the 500 Hz default PWM rate used to achieve that modulation effect. The issue might be alleviated by updating the firmware to increase the clock rate to the timer counters of the controller to achieve higher pulse rates (up to 31.25 kHz when operating from the internal 8 MHz clock) [49]. However, it is probably preferable to drive LEDs with a high-frequency switched-mode current driver (i.e., 1 MHz MCP1643, Microchip Technology) to achieve greater energy efficiency and eliminate large amplitude/low-frequency pulsing. Where designs require modulation of light intensity (i.e., for experimental trials), continuously variable control can be achieved by floating the ground to the feedback network on the driver.

## 5. Conclusions

We developed a custom programmable LED array to evaluate different lighting conditions to improve insect trapping. We demonstrated that unmodulated UV light resulted in significant improvement in trap catches of CRB in the field, as well as of ACP in forced-choice experiments in a screened cage. Our experiments also indicated that improvement in CRB trap catch is highest when lights are placed in the upper panel of traps, consistent with a dorsal orientation of insects to light [31], making it more likely that insects would strike a trap when “attractive” light is placed higher in the trap. All longer wavelength light sources placed in the panel of traps resulted in suppression of CRB catch, suggesting an aversion to these light sources. However, illumination of the trap cup with an amber LED resulted in a statistically significant increase in CRB catch. Similar to CRB, ACP in forced-choice experiments strongly preferred UV-illuminated traps. However, blue, yellow, and amber illumination of ACP traps also resulted in small but significant improvements in catch.

Some hardware limitations were identified, limiting the energy efficiency of the experimental lighting system and potentially precluding the use of the device to investigate the effects of variable lighting intensity and modulation in improving trap catches of insects. We are currently designing a new UV lighting module to be more compact and energy-efficient for widespread deployment with panel traps to improve the delineation of CRB on Hawaii, Kauai, and Maui, as well as designing a new programmable array to address some of the limitations of the experiments described here.

## Figures and Tables

**Table 1:** One-way ANOVA for the effect of light treatments on CRB catch.

Source	SSE	ν/dof	MSE	F	*p* (>F)
light	44.5	15	2.96667	3.62	1.07998 × 10^−5^
error	196.5	240	0.81875		
total	241	255			

**Table 2:** One-way ANOVA for effects of trial week on CRB catch.

Source	SSE	ν/dof	MSE	F	*p* (>F)
week	26.625	15	1.575	1.74	0.0445
error	217.375	240	0.90573		
total	241	255			

**Table 3:** One-way ANOVA for effects of trap position on CRB catch.

Source	SSE	ν/dof	MSE	F	*p* (>F)
position	13	15	0.86667	0.91	0.5511
error	228	240	0.95		
total	241	255			

## Data Availability

Most of the underlying data from this work is distilled in the Supplementary Materials. Any additional data (i.e., specific dates and times of CRB catches, and trap images these data are inferred from) is available from the authors upon request.

## Supplementary Materials

The following supporting information can be downloaded at https://www.mdpi.com/article/10.3390/insects16020170/s1, Figure S1: Setup for recording LED spectra; Figure S2: Recorded spectra of LEDs; Figure S3: Recorded light signals in citrus canopy and corresponding FFTs; Figure S4: Forced choice results between top performing LEDs for ACP attraction; Figure S5: Forced choice results between fully illuminated (“solid”) and modulated lighting in ACP traps; Figure S6: Results of ACP capture in field tests with traps illuminated 2 h before dawn; Table S1: Summary LED data from spectrometer; Table S2: Results of forced choice experiments between different wavelength LEDs in ACP stem traps vs. unlit traps; Table S3: Forced choice results between top performing LEDs for ACP capture; Table S4: Tabular results of forced choice experiments between “solid”/fully illuminated UV (385 nm) and modulated UV in ACP stem traps; Table S5: Tabular stem trap ACP capture data for field test.
