# Supplementary material for: Programmable LED Array for Evaluating Artificial Light Sources to Improve Insect Trapping
Source: Insects. 2025 Feb 6;16(2):170. doi: 10.3390/insects16020170 (PMC11856566; doi:10.3390/insects16020170)
Supplement: Supplementary file 1 [file insects-16-00170-s001.zip › insects-3379914-supplementary.pdf]

# Supplemental Materials

1. Manual of operation, hardware design files, and source code / installation files for programmable lighting system:

   https://github.com/danielje/Smart-Trap-Programmable-Lighting-Array

2. Spectral characterization of LED array

   Spectra of individual fully illuminated LEDs in the array were recorded in a dark closet with a handheld spectrometer (MK350N, UPRtek, Zhunan Township, Taiwan), integrating over 200 ms with detector window placed 20 cm from the middle (UV) LED, both with and without the diffuser assembly used for CRB trap experiments (Figure S1).

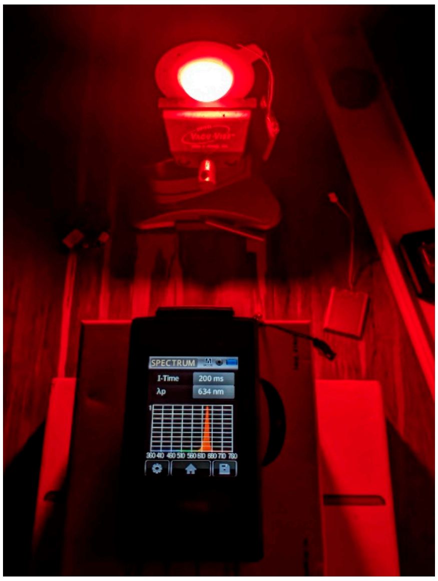


**Figure S1**. Setup for recording LED spectra (here measuring red LED through the diffuser used in CRB traps).

The recorded spectra of LEDs all powered with the same design current are illustrated in Figure S2, illustrating the well known “green-gap” where mid-wavelength LEDs near green operate with much lower quantum efficiencies than blue and red LEDs. Importantly, the diffuser does not appreciably alter the spectra of scattered light from any of the LEDs compared to directly measured LED light.

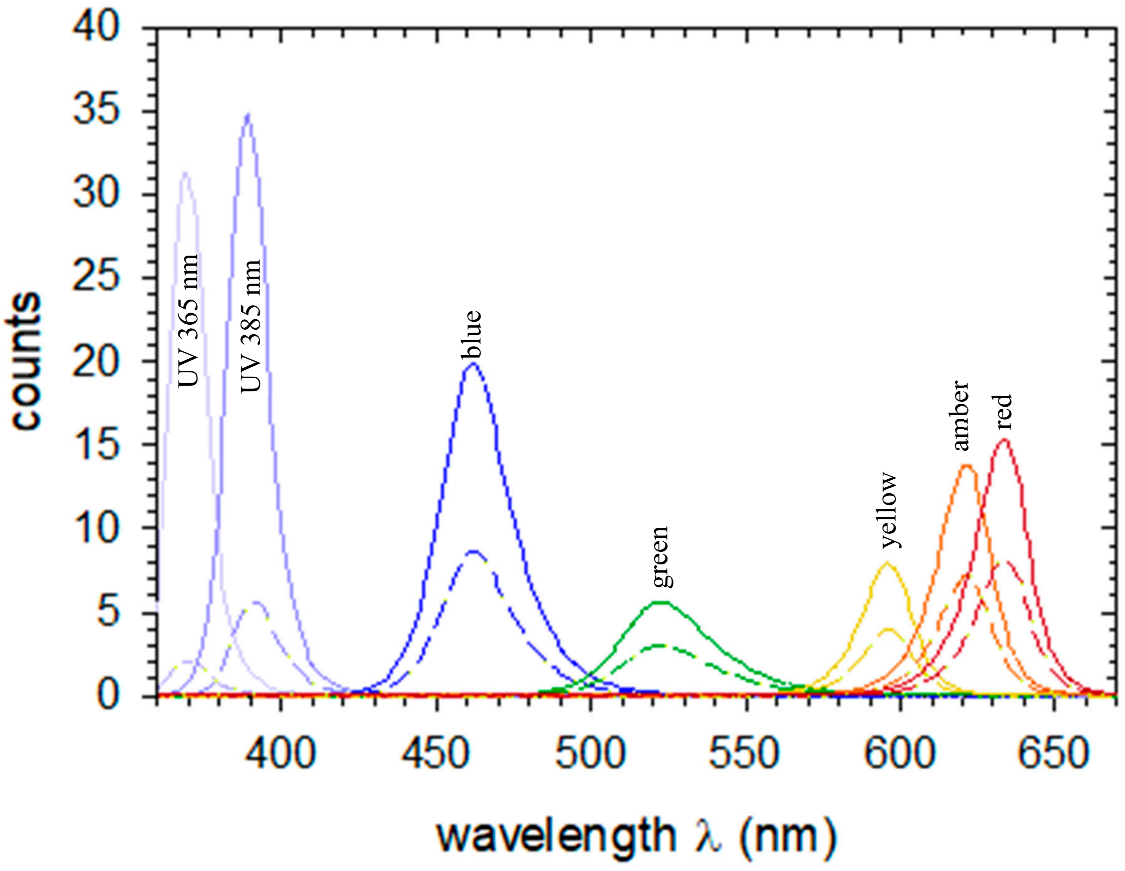


**Figure S2**. Recorded spectra of bare LEDs (solid lines) and corresponding spectra through diffuser used in CRB traps (dashed lines). "Counts" are the product of normalized spectral data recorded by instrument (normalized to 1 at the peak wavelength) and the "peak count" recorded by the instrument, as a better comparison of overall radiance of different color LEDs.

Summary data from the spectrometer (Table S1) also suggest very little spectral shift due to the diffuser, with larger transmission losses for shorter wavelength LEDs compared to those from green, yellow, amber and red. The illuminance values (in units of lux) recorded by the instrument are not useful for comparing total LED radiance for different colors, as it is heavily weighted to green and excludes UV- i.e. the green LED resulted in the highest illuminance despite having the lowest peak count, and UV LEDs resulted in virtually no "illuminance".

**Table S1**. Summary LED data from spectrometer. Ratio of values recorded through the diffuser normalized to the corresponding values recorded directly from the LED (last column) indicate little to no spectral shift due to the diffuser, and larger transmission losses through the diffuser for the shorter wavelength LEDs compared to green, yellow, amber, and red LEDs.

| | | Bare | Diffuser | Ratio (D/B)[*] |
|---|---|---|---|---|
| | $\lambda_p$ (nm)[†] | 369 | 370 | 1.0027 |
| **UV 365 nm** | peak count[‡] | 31.33 | 2.10 | 0.0671 |
| | ∫ count dλ[§] | 486.21 | 40.52 | 0.0833 |
| | Lux[¶] | 1 | 1 | 1.0000 |
| | $\lambda_p$ (nm)[†] | 389 | 391 | 1.0051 |
| **UV 385 nm** | peak count[‡] | 34.84 | 5.61 | 0.1610 |
| | ∫ count dλ[§] | 615.29 | 114.27 | 0.1857 |
| | Lux[¶] | 0 | 0 | nan |
| | $\lambda_p$ (nm)[†] | 462 | 463 | 1.0022 |
| **blue** | peak count[‡] | 19.85 | 8.62 | 0.4345 |
| | ∫ count dλ[§] | 606.91 | 267.97 | 0.4415 |
| | Lux[¶] | 38 | 17 | 0.4474 |
| | $\lambda_p$ (nm)[†] | 523 | 525 | 1.0038 |
| **green** | peak count[‡] | 5.63 | 3.02 | 0.5353 |
| | ∫ count dλ[§] | 231.12 | 126.49 | 0.5473 |
| | Lux[¶] | 115 | 62 | 0.5391 |
| | $\lambda_p$ (nm)[†] | 596 | 596 | 1.0000 |
| **yellow** | peak count[‡] | 7.93 | 3.98 | 0.5022 |
| | ∫ count dλ[§] | 184.95 | 95.47 | 0.5162 |
| | Lux[¶] | 85 | 43 | 0.5059 |
| | $\lambda_p$ (nm)[†] | 622 | 622 | 1.0000 |
| **amber** | peak count[‡] | 13.82 | 7.18 | 0.5191 |
| | ∫ count dλ[§] | 331.64 | 174.19 | 0.5252 |
| | Lux[¶] | 88 | 45 | 0.5114 |
| | $\lambda_p$ (nm)[†] | 634 | 634 | 1.0000 |
| **red** | peak count[‡] | 15.40 | 8.15 | 0.5295 |
| | ∫ count dλ[§] | 373.47 | 200.89 | 0.5379 |
| | Lux[¶] | 68 | 36 | 0.5294 |

[*] Corresponding value with diffuser normalized to value without diffuser.

[†] Wavelength of peak intensity.

[‡] Integral of spectrometer count over entire recorded spectra (proxy for total radiance from LED).

[¶] Instrument illuminance reading (in units of lux). Lux is not a good representation for total LED radiance, as it is weighted to coincide with human light sensitivity, with heaviest weight in green, and no weight outside of “visible” wavelengths including UV.

3. Selection of modulation frequency to test in ACP traps

To choose a modulation frequency to test the effects of the simple modulation schemes (pulsed, or sinusoidal half waves) for the LED array we used a broad spectrum clear lensed photodiode (SFH 213, ams-Osram, Premstätten, Austria) oriented downwards in a citrus canopy, interfaced in a “2 electrode” configuration to an open source potentiostat, sampling photocurrent at 100 Hz with no bias for intervals of at least 21 s. Samples were background corrected to remove the (predominant) DC component of the light, and then transformed to the frequency domain using a 2048 point Fast Fourier Transform (example data in Figure S3). We somewhat arbitrarily selected 1.4 Hz, as a frequency corresponding to regularly observed strong peaks above 1 Hz.

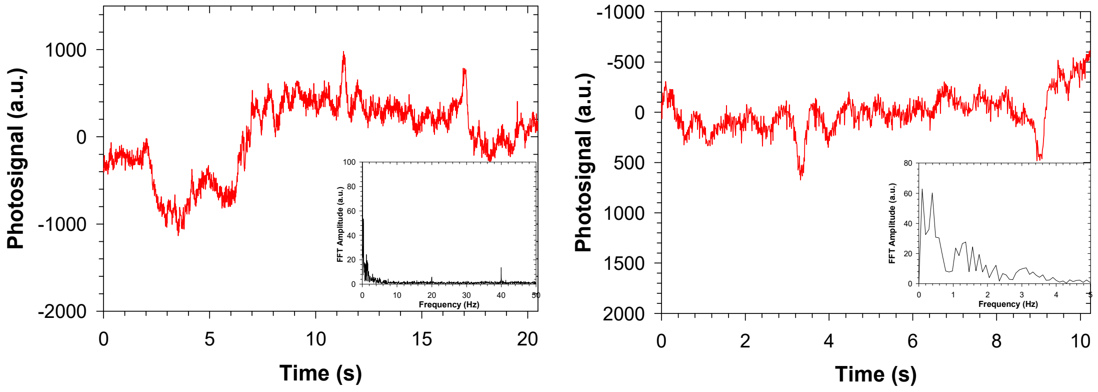


**Figure S3.** Recorded light signals observed in citrus canopy, and corresponding FFTs.

4. ACP Forced choice experiments between traps with different lighting conditions.

**Table S2**. Results of forced choice experiments between different wavelength LEDs in ACP stem traps vs. unlit traps.

| | ACP catches in | |
|---|---|---|
| | lighted trap | unlit trap |
| UV | 16 | 0 |
| UV | 2 | 0 |
| UV | 7 | 0 |
| Blue | 5 | 0 |
| Blue | 5 | 2 |
| Blue | 4 | 0 |
| Green | 2 | 2 |
| Green | 3 | 3 |
| Green | 3 | 2 |

| | ACP catches in | |
|---|---|---|
| | lighted trap | unlit trap |
| Yellow | 4 | 0 |
| Yellow | 1 | 0 |
| Yellow | 4 | 0 |
| Yellow | 2 | 0 |
| Amber | 0 | 0 |
| Amber | 6 | 0 |
| Amber | 0 | 0 |
| Red | 2 | 1 |
| Red | 1 | 3 |
| Red | 0 | 3 |

**Table S3**. Forced choice results between top performing LEDs (UV 385, Blue, Yellow; fully illuminated).

| | | ACP Catch | | pooled p |
|---|---|---|---|---|
| Test | Control | Test | Control | catch≥observed |
| UV | Blue | 14 | 5 | |
| UV | Blue | 23 | 2 | 2.78E-10 |
| UV | Blue | 19 | 1 | |
| UV | Yellow | 8 | 4 | |
| UV | Yellow | 16 | 4 | 3.08E-05 |
| UV | Yellow | 14 | 2 | |
| Blue | Yellow | 7 | 3 | |
| Blue | Yellow | 8 | 9 | 1.62E-01 |
| Blue | Yellow | 7 | 3 | |

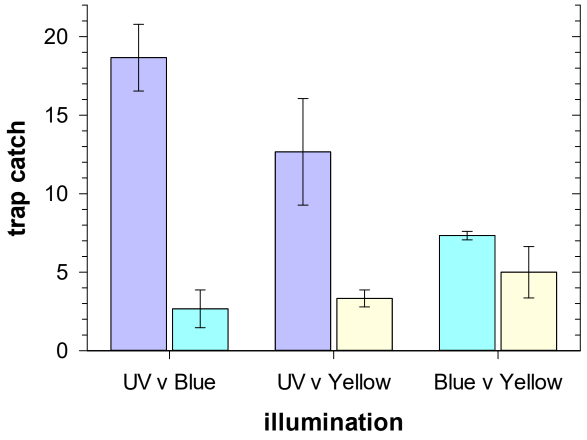


**Figure S4**. Graphical summary of Table S3 data.

**Table S4**. Tabular results of forced choice experiments between "solid" / fully illuminated UV (385 nm) and modulated (1.4 Hz) UV (385 nm) stem traps for ACP.

| | | ACP Catch | | Pooled p |
|---|---|---|---|---|
| Test | Control | Test | Control | catch ≥ observed |
| Solid | Wave | 8 | 10 | |
| Solid | Wave | 16 | 1 | 1.33E-03 |
| Solid | Wave | 15 | 5 | |
| Solid | Pulse | 5 | 10 | |
| Solid | Pulse | 10 | 4 | 2.20E-01 |
| Solid | Pulse | 9 | 4 | |

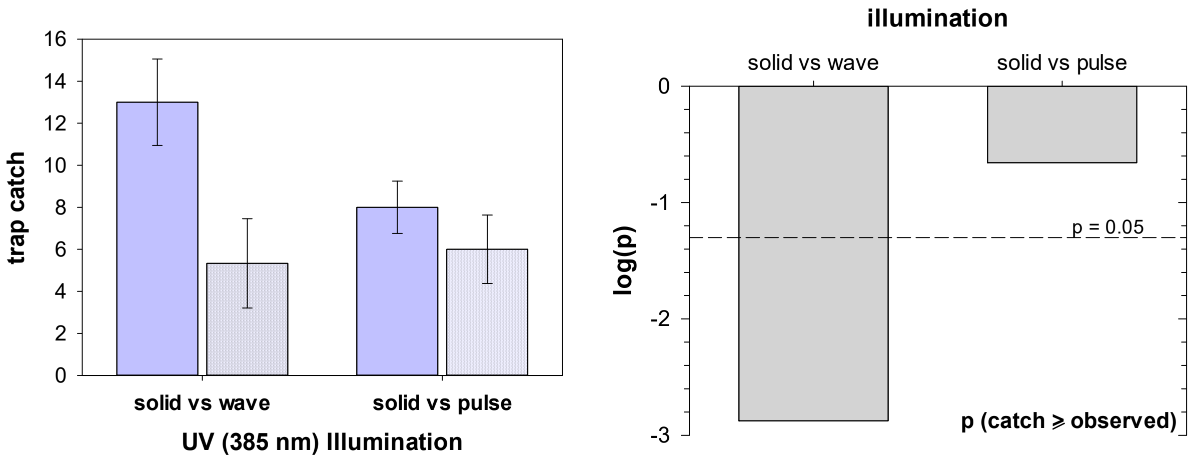


**(a)** **(b)**

**Figure S5**. Results of forced choice between fully illuminated ("solid") and modulated lighting ACP traps (a), and binomial probabilities of observed catch or greater in fully illuminated trap assuming both treatments are equal (b)

5. ACP field data with traps illuminated with different LEDs at 64% intensity.

**Table S5**. Tabular stem trap ACP capture data for field test with (daily catch data with different wavelength LEDs illuminated 64% intensity for 2 hours before sunrise).

| **LED** | Day 1 | Day 2 | Day 3 | Day 4 | Day 5 | Day 6 | **Total** |
|---|---|---|---|---|---|---|---|
| **UV 385nm** | 1 | 0 | 1 | 0 | 3 | 1 | **6** |
| **Blue** | 1 | 0 | 0 | 0 | 0 | 0 | **1** |
| **Green** | 0 | 0 | 1 | 1 | 2 | 0 | **4** |
| **Yellow** | 0 | 0 | 0 | 0 | 1 | 1 | **2** |
| **Amber** | 0 | 1 | 1 | 1 | 2 | 0 | **5** |
| **No Light** | 1 | 1 | 1 | 4 | 0 | 1 | **8** |

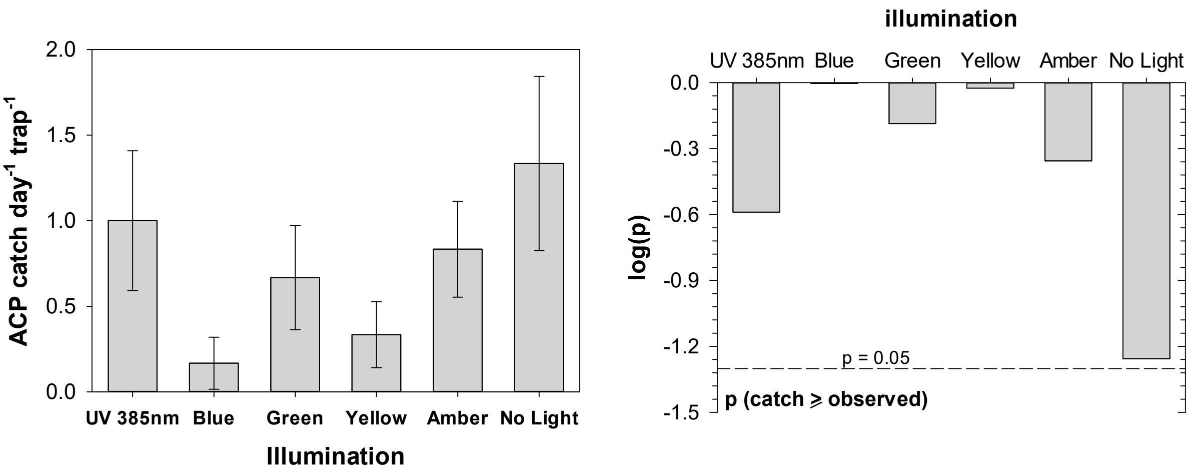


(a) (b)

**Figure S6**. ACP catches in traps illuminated with different wavelength LEDs two hours before sunrise (and one unlit trap), rotated daily through 6 stations around an ACP infested mock orange hedge (a), and binomial probabilities of observed catch or more in each treatment assuming all treatments are equal (b)
